# Effects of testicular dysgenesis syndrome components on testicular germ cell tumor prognosis and oncological outcomes

**DOI:** 10.1590/S1677-5538.IBJU.2019.0387

**Published:** 2020-07-31

**Authors:** Ismail Selvi, Erdem Ozturk, Taha Numan Yikilmaz, Selcuk Sarikaya, Halil Basar

**Affiliations:** 1 Karabük University Training and Research Hospital Department of Urology Karabük Turkey Department of Urology, Karabük University Training and Research Hospital, Karabük, Turkey; 2 Health Science University Dr. Abdurrahman Yurtaslan Ankara Oncology Training and Research Hospital Department of Urology Ankara Turkey Department of Urology, Health Science University Dr. Abdurrahman Yurtaslan Ankara Oncology Training and Research Hospital, Ankara, Turkey; 3 Health Science University Gulhane Training and Research Hospital Department of Urology Ankara Turkey Department of Urology, Health Science University Gulhane Training and Research Hospital, Ankara, Turkey

**Keywords:** Cryptorchidism, Testicular Germ, Cell Tumor [Supplementary Concept], Hypospadias

## Abstract

**Purpose:**

To evaluate whether components of Testicular Dysgenesis Syndrome (TDS) affect testicular germ cell tumor (TGCT) prognosis and oncological outcomes. According to the hypothesis called TDS; undescended testis, hypospadias, testicular cancer and spermatogenic disorders share the same risk factors and have a combined fetal origin.

**Materials and Methods:**

We retrospectively evaluated the stages and oncological outcomes of 69 patients who underwent radical orchiectomy between January 2010 and December 2014 due to TGCT in our department. The presence of undescended testis, hypospadias and semen parameters disorders were recorded according to anamnesis of patients.

**Results:**

Among 69 patients with TGCT, only 16 (23.1%) had TDS. Significantly higher rate of TDS (36.1% vs. 9.1%) was observed at the advanced stages of TGCT(p=0.008). In the TDS group, the rates of local recurrence (50% vs. 11.3%, p<0.001), distant metastasis (93.6% vs. 3.8%, p<0.001) and cancer-spesific mortality (87.5% vs. 3.8%, p<0.001) were found significantly higher than those without TDS. The predicted time for recurrence-free survival (13.70±5.13 vs. 100.96±2.83 months, p<0.001) metastasis-free survival (13.12±4.21 vs. 102.79±2.21 months, p <0.001) and cancer-specific survival (13.68±5.38 vs. 102.80±2.19 months, p<0.001) were also statistically lower in this group.

**Conclusions:**

According to our preliminary results, there is an apparent relationship between TDS and tumor prognosis. Even if the components of TDS alone did not contain poor prognostic features for TGCT, the presence of TDS was found as the most important independent predictive factor for oncological outcomes in both seminomas and nonseminomas as well as all patients with TGCT.

## INTRODUCTION

Testicular dysgenesis syndrome (TDS) is one of the current topics that has been described in recent years. Undescended testis, hypospadias, decreased spermatogenesis and testicular germ cell tumor (TGCT) form TDS components (1). One or more of these disorders occur in about 1 in 6 young men in Northern Europe (2). TDS has a common fetal origin associated with deficiencies in fetal androgen production (3). A failure in normal differentiation of fetal germ cells is effective in the formation of this syndrome. Increase in the incidence of TGCT in young men is also related to this mechanism. That is why TDS have been associated with TGCT (1). The hypotheses related to TDS have been strengthened by new studies since the last two decades (4, 5). Althought there are still controversial views about TDS, these studies have aimed to provide evidence verifying the reality of TDS based on a few key aspects, such as genetic factors, environmental endocrine-disrupting chemicals, lifestyle factors and intrauterine growth disorders (6, 7).

The biological mechanism of TDS was tried to be demonstrated in animal models due to limitations in human studies (4). Nevertheless, more evidence is needed to reinforce TDS hypothesis (8). According to the literature, semen analysis and testicular histology support the association between TGCT and TDS (9). But there is no detailed evaluation to show the effects of TDS components on TGCT prognosis. We aimed to evaluate whether components of TDS have an effect on TGCT prognosis and oncological outcomes.

## MATERIALS AND METHODS

After obtaining the approval of the local ethics committee (protocol number: 77192459-050.99-E.2812, 3/19), we retrospectively evaluated the stages and oncological outcomes of 77 patients who underwent radical orchiectomy between January 2010 and December 2014 due to TGCT at our department. The presence of undescended testis, hypospadias, disorders of semen parameters and atrophic testis (testicular volume <12mL) were recorded. As our study also included non-married patients, it was not possible to evaluate the fertility status for all patients. Instead of this, disorders of semen parameters were examined. Demographic data, histological tumor types, clinical stages, tumor side, tumor sizes, expression of serum tumour markers (Alpha-fetoprotein, Beta human chorionic gonadotropin [β-hCG] and Lactate dehydrogenase [LDH]), prognostic factors in pathology specimen, post-orchiectomy follow-up period, presence of adjuvant therapy after orchiectomy, rates of local recurrence, distant metastasis and cancer-specific survival (CSS) were also recorded. 69 patients with complete data were included in the study. The patients whose data could not be completely collected were excluded from the study.

Tumor stages were recorded according to the 2009 classification of Tumor-Node-Metastasis. Patients were divided into two main groups. Stage IA and IB were determined as early stage (Group I). Stage IS, IIA/IIB/IIC and IIIA/IIIB/IIIC were determined as advanced stage (Group II).

The definition of TDS involves the presence of at least two of the following: undescended testis, hypospadias, decreased spermatogenesis and testicular germ cell tumor (4). As all patients had TGCT, those with any of undescended testis, hypospadias or disorders of semen parameters formed the TDS group. 16 patients with TDS and 53 patients without TDS were determined and a subgroup analysis was also done.

Pathological prognostic factors were based on the Guidelines of European Association of Urology on Testicular Tumors (9).The prognostic factors for the stage I seminomas were rete testis involvement and tumor size greater than 4cm.The presence of lymphovascular invasion (LVI), the percentage of embryonal carcinoma more than 50% and the proliferation rate above 70% were prognostic factors for stage I non-seminomas.

### Statistical analysis

To compare the differences between the two groups, the normality status was evaluated by Kolmogorov-Smirnov and Shapiro-Wilk tests. Pearson Chi-square or Fisher exact analysis for categorical variables, Mann-Whitney U test for continuous variables in non-normal distribution were used. Kaplan-Meier was used for survival analysis and Cox regression analysis was used for determining the independent variables. The analyzes were performed using IBM SPSS Statistics 21 (IBM, Armonk, NY USA) software. P <0.05 was considered statistically significant.

## RESULTS

Median age of the 69 male patients was 31 (min:8-max:60). Demographic and clinical characteristics of the patients are shown in Tables 1 and 2. During the median follow-up period of 57 (6–106) months, the distant metastases were located at lung in 8 patients, liver in 4 patients and non-regional lymph nodes in 5 patients.

**Table 1:** Distribution of patients according to tumor stages, histologic tumor types, components of testicular dysgenesis syndrome and oncologic outcomes.

Stage	Number of seminoma patients	Number of nonseminoma patients	Number of mix germ cell tumor	History of undescended testis	History of hypospadiass	History of subfertility	Numbers of patients with testicular dysgenesis syndrome	Post-treatment recurrence and histological subtype	Presence of testicular dysgenesis syndrome in patients with recurrence	Post-treatment metastasis and histological subtype	Presence of testicular dysgenesis syndrome in patients with metastasis	Post-treatment mortality and histological subtype	Presence of testicular dysgenesis syndrome in deceased patients
**IA**	11	4	5	-	-	-	0 (% 0)	0	0 (% 0)	1 (NS)	0 (%0)	1 (NS)	0 (%0)
**IB**	8	4	1	2	-	2	3 (% 23)	2 (NS)	1 (% 50)	1 (M),1(S)	2 (%100)	1 (M),1(S)	2 (%100)
**IS**	1	1	2	-	-	-	0 (% 0)	1 (M)	0 (% 0)	0		0	
**IIA**	0	1	0	-	-	-	0 (% 0)	0	0 (% 0)	0		0	
**IIB**	0	3	1	1	-	1	1 (% 25)	1 (NS)	0 (% 0)	1 (NS)	1 (%100)	1 (NS)	1(%100)
**IIC**	3	0	0	1	1	-	1 (% 33.3)	1 (S)	1 (% 100)	2 (S)	1 (%50)	2 (S)	1(%50)
**IIIA**	1	2	0	-	-	1	1 (% 33.3)	1 (NS)	0 (% 0)	1 (NS)	1 (%100)	0	
**IIIB**	5	3	1	2		2	4 (% 44.4)	2 (NS), 1 (S)	2 (% 66.6)	2(S), 2(NS)	4 (%100)	2(S), 2(NS)	4(%100)
**IIIC**	6	6	0	3	2	3	6 (% 50)	2 (S), 3 (NS)	4 (% 80)	3(NS), 3(S)	6 (%100)	3(NS), 3(S)	6(%100)
**Total number**	**35**	**24**	**10**	**9**	**3**	**9**	**16 (% 23.1)**	**14**	**8 (% 57.1)**	**17**	**15 (%88.2)**	**16**	**14(%87.5)**

**S** = Seminoma, **NS** = Non-seminoma; **M** = Mix germ cell tumor

**Table 2:** Demographic, pathological, clinical data and oncologic outcomes of the patients.

Parameters		Group I	Group II	Total	p value
		(Early stage TGCT) (n:33)	(Advanced stage TGCT) (n:36)	(n:69)	
**Age**					
	Median (25^th^-75^th^percentiles)	31.00 (27.00-37.00)	30.00 (24.25-41.75)	31 (25-40)	† 0.709
**Tumor size (cm)**					
	Median (25^th^-75^th^percentiles)	3.50 (2.15-4.55)	5.55 (3.52-7.20)	4.20 (2.65-6.50)	† 0.002*
**Tumor laterality (n,%)**					
	Left	10 (30.3)	13 (36.1)	23 (33.3)	† 0.877
	Right	21 (63.6)	21 (58.3)	42 (60.9)	
	Bilateral	2 (6.1)	2 (5.6)	4 (5.8)	
**Histopathological subtype(n,%)**					
	Seminoma	19 (57.6)	16 (44.4)	35 (50.7)	
	Non-seminoma	8 (24.2)	16 (44.4)	24 (34.8)	† 0.202
	Mix	6 (18.2)	4 (11.1)	10 (14.5)	
**AFP (ng/mL)**					
	Median (25^th^-75^th^percentiles)	5.00 (1.70-10.70)	6.90 (2.75-354.25)	5.50 (2.15-74.37)	† 0.058
**β-hCG (mIU/mL)**					
	median (25^th^-75^th^percentiles)	4.90 (1.30-33.30)	62.10 (5.95-911.02)	15.20 (2.50-128.00)	† 0.005*
**LDH (U/l)**					
	Median (25^th^-75^th^percentiles)	208.00 (155.00266.00)	717.00 (330.00-1299.25)	309.00 (202.00740.00)	†<0.001*
**ITGCN (n,%)**					
	Present	15 (45.5)	20 (55.6)	35 (50.7)	‡ 0.402
	Absent	18 (54.5)	16 (44.4)	34 (49.3)	
***Rete testis* involvement (n,%)**					
	Present	8 (24.2)	7 (19.4)	15 (21.7)	‡ 0.629
	Absent	25 (75.8)	29 (80.6)	54 (78.3)	
**Tumor diameter> 4 cm (n,%)**					
	Yes	12 (36.4)	24 (66.7)	36 (52.2)	‡ 0.012*
	No	21 (63.6)	12 (33.3)	33 (47.8)	
**Lymphovascular invasion (n,%)**					
	Present	7 (21.2)	20 (55.6)	27 (39.1)	‡ 0.004*
	Absent	26 (78.8)	16 (44.4)	42 (60.9)	
**Embryonal carcinoma rate >50% (n,%)**					
	Present	7 (21.2)	12 (33.3)	19 (27.5)	‡ 0.260
	Absent	26 (78.8)	24 (66.7)	50 (72.5)	
**Proliferation rate > 70% (n,%)**					
	Present	3 (9.1)	7 (19.4)	10 (14.5)	‡ 0.222
	Absent	30 (90.9)	29 (80.6)	59 (85.5)	
**Undescended testis (n,%)**					
	Present	2 (6.1)	7 (19.4)	9 (13.0)	‡ 0.099
	Absent	31 (93.9)	29 (80.6)	60 (87.0)	
**Disorders of semen parameters(n,%)**					
	Present	2 (6.1)	7 (19.4)	9 (13.0)	‡ 0.099
	Absent	31 (93.9)	29 (80.6)	60 (87.0)	
**Hypospadias (n,%)**					
	Present	0 (0.0)	3 (8.3)	3 (4.3)	§ 0.240
	Absent **Atrophic testis (n,%)**	33 (100.0)	33 (91.7)	66 (95.7)	
	Present	1 (3.0)	6 (16.7)	7 (10.1)	‡ 0.061
	Absent	32 (97.0)	30 (83.3)	62 (89.9)	
**Presence of TDS (n,%)**					
	Present	3 (9.1)	13 (36.1)	16 (23.2)	‡ 0.008*
	Absent	30 (90.9)	23 (63.9)	53 (76.8)	
	Local recurrence rate (n,%)	2 (6.1)	12 (33.3)	14 (20.3)	‡ 0.015*
	Distant metastasis rate (n,%)	3 (9.1)	14 (38.9)	17 (24.6)	‡ 0.004*
	Cancer-specific survival rate (%)	90.9	63.9	76.8	‡ 0.008*

* = p <0.05 Asteriks (*) indicates statistical significance; **AFP**: alpha-fetoprotein; **β-hCG** = beta human chorionic gonadotropin; **ITGCN** = Intratubular germ cell neoplasia; **LDH** = lactate dehydrogenase; **TDS** = Testicular dysgenesis syndrome; **TGCT** = testicular germ cell tumor

† = Mann-Whitney U test

‡ = Chi-square test

§ = Fisher’s Exact test

When the early and advanced tumor stages were compared, it was shown that the predicted time for recurrence-free survival (RFS) (71.61±8.02 vs. 96.57±4.65 months, p=0.01), metastasis-free survival (MFS) (68.32±7.91 vs. 96.99±4.43 months, p=0.003) and cancer-specific survival CSS (71.18±7.73 vs. 96.67±4.57 months, p=0.007) were statistically lower in patients with advanced stage (Figures 1A, 1B and 1C).

**Figure 1A:**
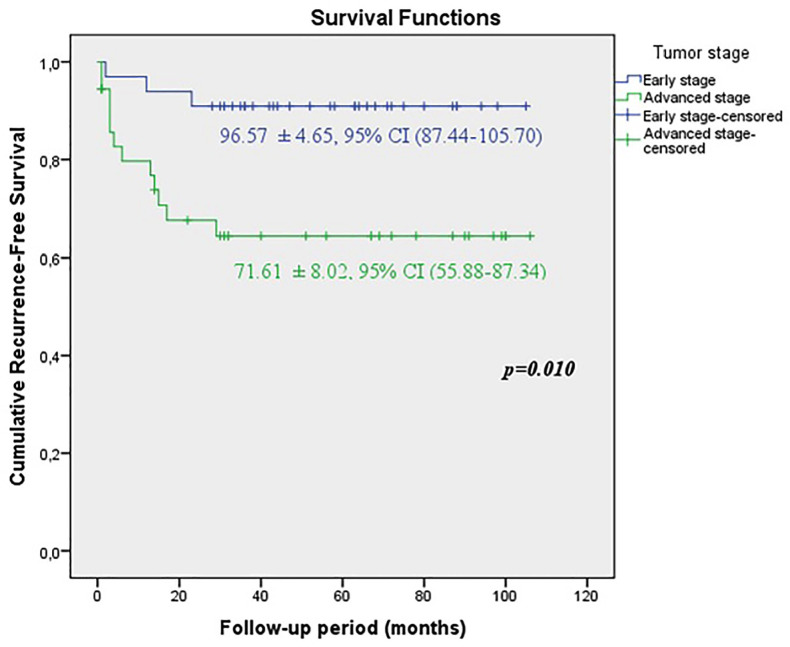
KKaplan-Meier plots of recurrence-free survival according to the early and advanced stages for all tumors.

**Figure 1B:**
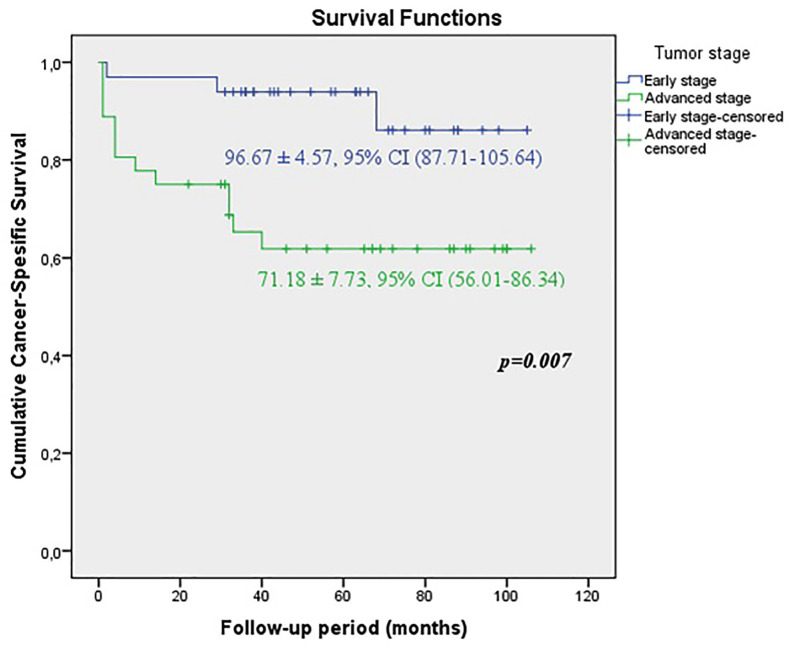
Kaplan-Meier plots of cancer-specific survival according to the early and advanced stages for all tumors.

**Figure 1C:**
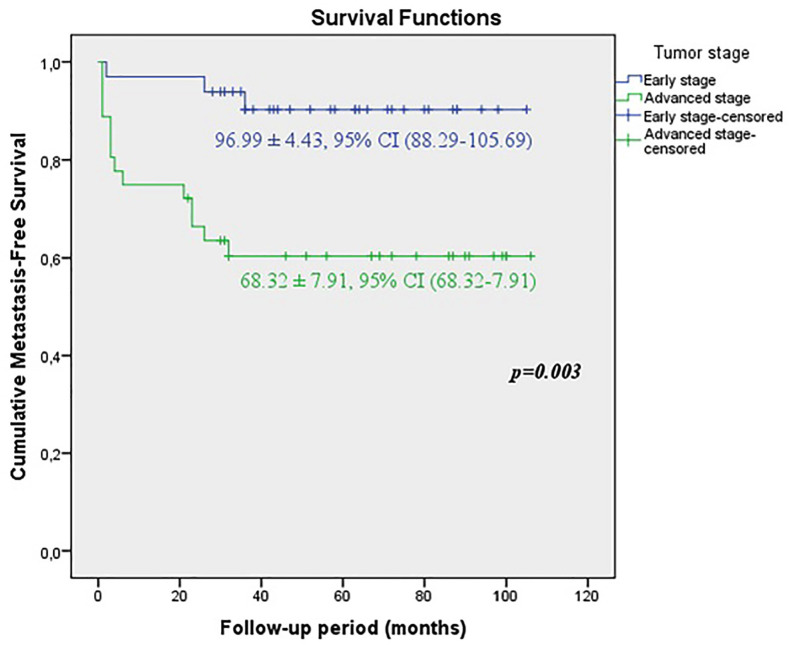
Kaplan-Meier plots of metastasis-free survival according to the early and advanced stages for all tumors.

In a subgroup analysis, patients were classified in terms of the presence of TDS. Significantly higher TDS rates (36.1% vs. 9.1%) were observed in the advanced stages (p=0.008) (Table-2). In the TDS group, the rates of loctal recurrence (50% vs. 11.3%, p <0.001), distant metastasis (93.6% vs. 3.8%, p <0.001) and cancer-specific mortality (87.5% vs. 3.8%, p <0.001) were found significantly higher than those without TDS (Table-3). When patients with seminoma and non-seminoma were compared between themselves, in the presence of TDS, the rate of local recurrence (88.9% vs. 57.1%) was higher in non-seminomas, whereas distant metastasis (100% vs. 88.9%) and cancer-specific mortality rates (100% vs. 77.8%) were higher in seminomas (Table-3).

**Table 3:** Oncologic outcomes of the patients in terms of testicular dysgenesis syndrome.

All patients with testicular germ cell tumor		Patients with TDS (n:16)	Patients without TDS (n:53)	Total (n:69)	p value
**Local recurrence(n,%)**					
	Present	12 (75.0)	3 (5.7)	16 (23.2)	‡<0.001*
	Absent	4 (25.0)	50 (94.3)	53 (76.8)	
**Distant metastasis (n,%)**					
	Present	15 (93.6)	2 (3.8)	17 (24.6)	‡<0.001*
	Absent	1 (6.3)	51 (96.2)	52 (75.4)	
**Cancer spesific mortality (n,%)**					
	Present	14 (87.5)	2 (3.8)	16 (23.2)	‡<0.001*
	Absent	2 (12.5)	51 (96.2)	53 (76.8)	
**Patients with seminoma**		Patients with TDS (n:7)	Patients without TDS (n:28)	Total (n:35)	p value
**Local recurrence(n,%)**					
	Present	4 (57.1)	1 (3.6)	5 (14.3)	§ 0.003*
	Absent	3 (42.9)	27 (96.4)	30 (85.7)	
**Distant metastasis (n,%)**					
	Present	7 (100.0)	1 (3.6)	8 (22.9)	§ <0.001*
	Absent	0 (0.0)	27 (96.4)	27 (77.1)	
**Cancer specific mortality (n,%)**					
	Present	7 (100.0)	1 (3.6)	8 (22.9)	§ <0.001*
	Absent	0 (0.0)	27 (96.4)	27 (77.1)	
**Patients with non-seminoma**		Patients with TDS (n:9)	Patients without TDS (n:25)	Total (n:34)	p value
**Local recurrence(n,%)**					
	Present	8 (88.9)	1 (4.0)	9 (26.5)	§ <0.001*
	Absent	1 (11.1)	24 (96.0)	25 (73.5)	
**Distant metastasis (n,%)**					
	Present	8 (88.9)	1 (4.0)	9 (26.5)	§ <0.001*
	Absent	1 (11.1)	24 (96.0)	25 (73.5)	
**Cancer specific mortality (n,%)**					
	Present	7 (77.8)	1 (4.0)	8 (23.5)	§ <0.001*
	Absent	2 (22.2)	24 (96.0)	26 (76.5)	

* = p <0.05 Asteriks (*) indicates statistical significance.

**TDS** = Testicular dysgenesis syndrome

‡ = Chi-square test

§ = Fisher’s Exact test

The predicted time for recurrence-free survival (RFS) (13.70±5.13 vs.100.96±2.83 months, p <0.001), metastasis-free survival (MFS) (13.12±4.21 vs. 102.79±2.21 months, p <0.001) and cancer-specific survival (CSS) (13.68±5.38 vs. 102.80±2.19 months, p <0.001) were statistically lower in patients with TDS (Figures 2A, 2B and 2C). In the presence of TDS, the predicted time for RFS was longer in patients with seminoma (13.64±4.56 vs.12.66±6.48 months, p <0.001). Conversely, the predicted time for MFS (9.14±4.40 vs. 16.22±6.59 months, p <0.001) and CSS (11.71±5.16 vs. 24.11±8.39 months, p <0.001) were statistically shorter in patients with seminoma (Figures 3A, 3B, 3C and Figures 4A, 4B, 4C).

**Figure 2A:**
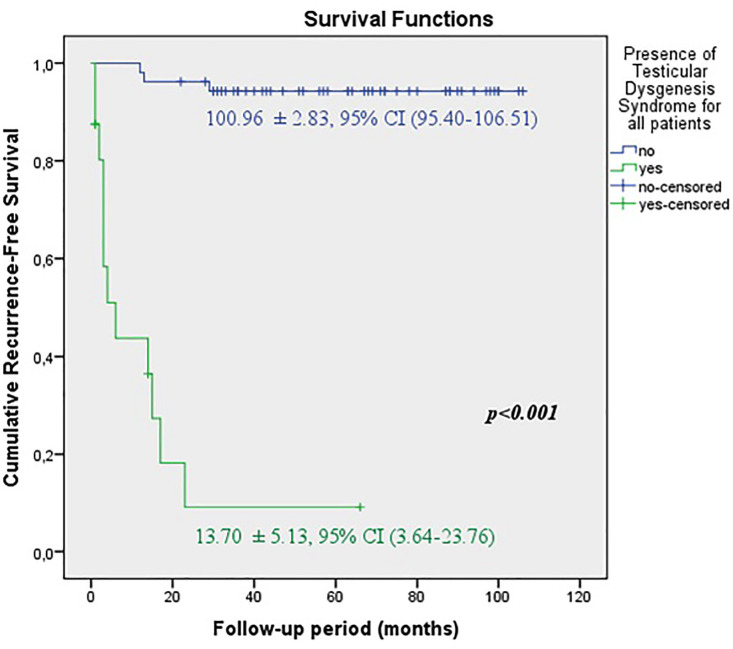
Kaplan-Meier plots of recurrence-free survival according to presence of Testicular Dysgenesis Syndrome for all patients.

**Figure 2B:**
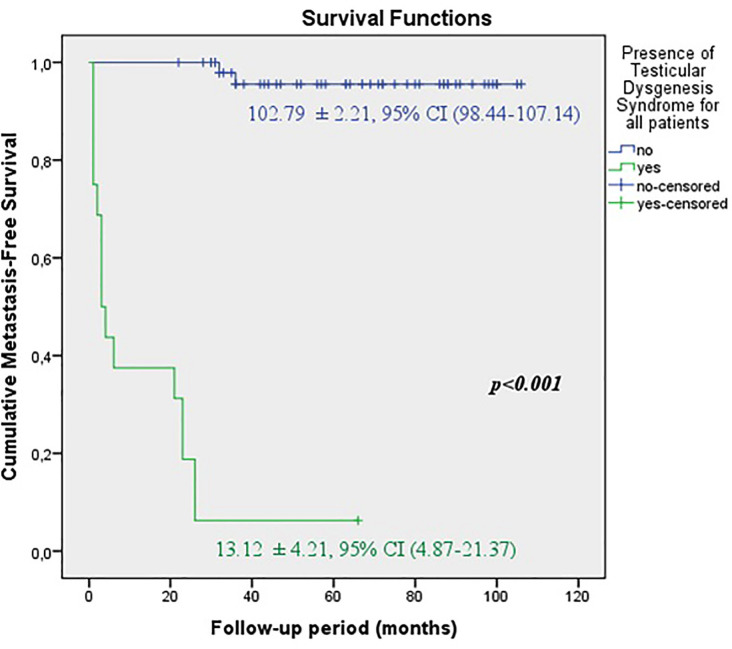
Kaplan-Meier plots of metastasis-free survival according to presence of Testicular Dysgenesis Syndrome for all patients.

**Figure 2C:**
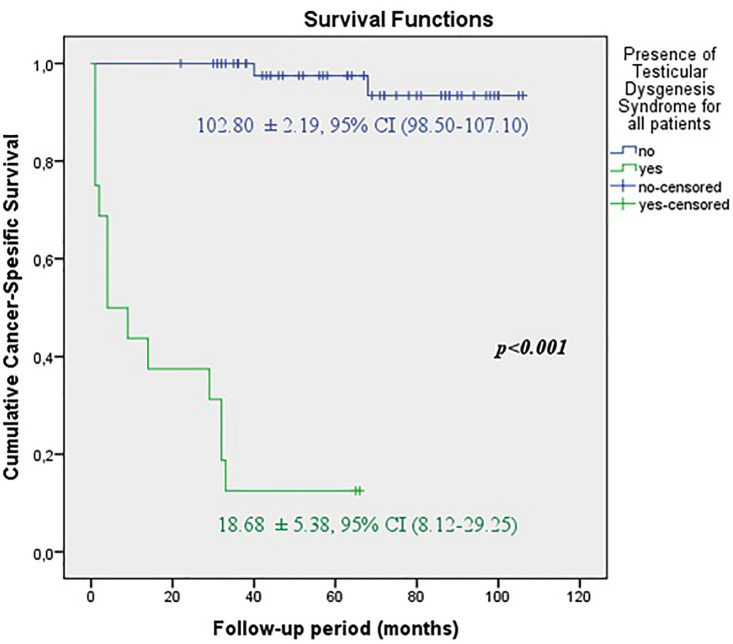
Kaplan-Meier plots of cancer-specific survival according to presence of Testicular Dysgenesis Syndrome for all patients.

**Figure 3A:**
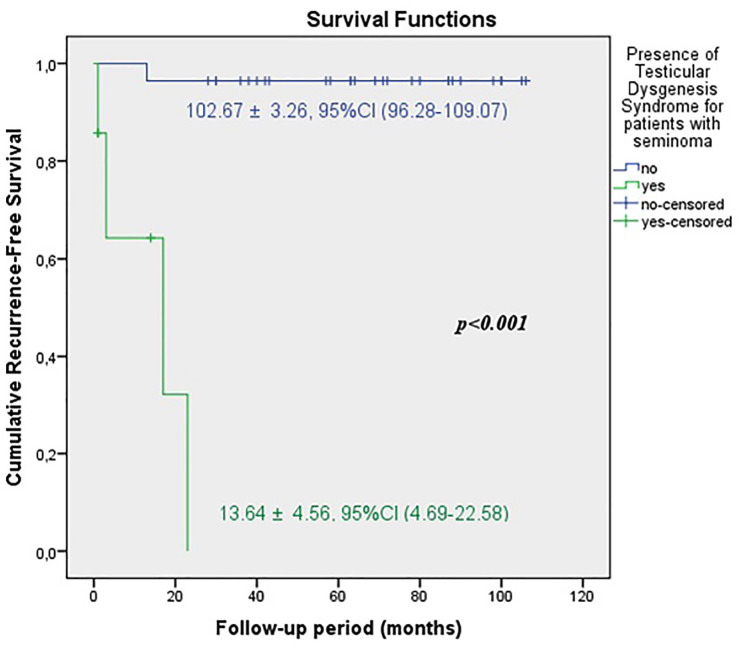
Kaplan-Meier plots of recurrence-free survival according to presence of Testicular Dysgenesis Syndrome for patients with seminoma.

**Figure 3B:**
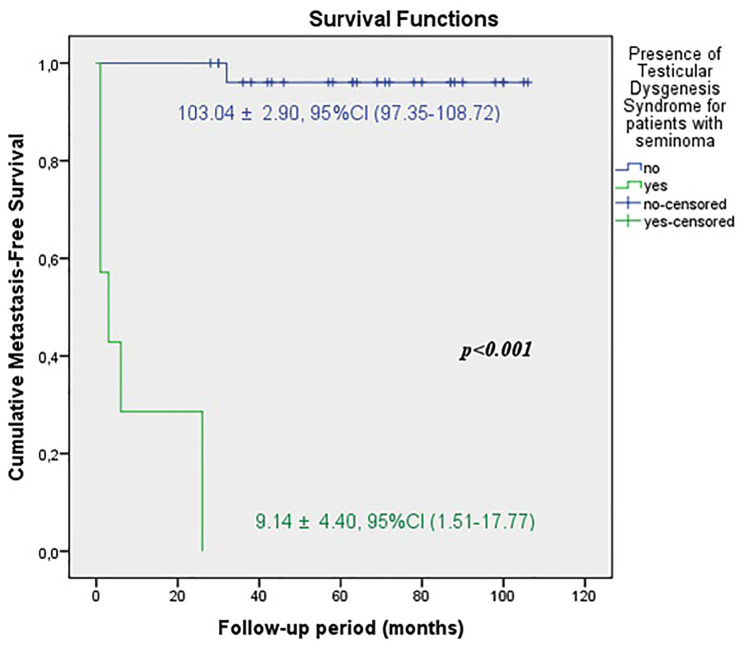
Kaplan-Meier plots of metastasis-free survival according to presence of Testicular Dysgenesis Syndrome for patients with seminoma.

**Figure 3C:**
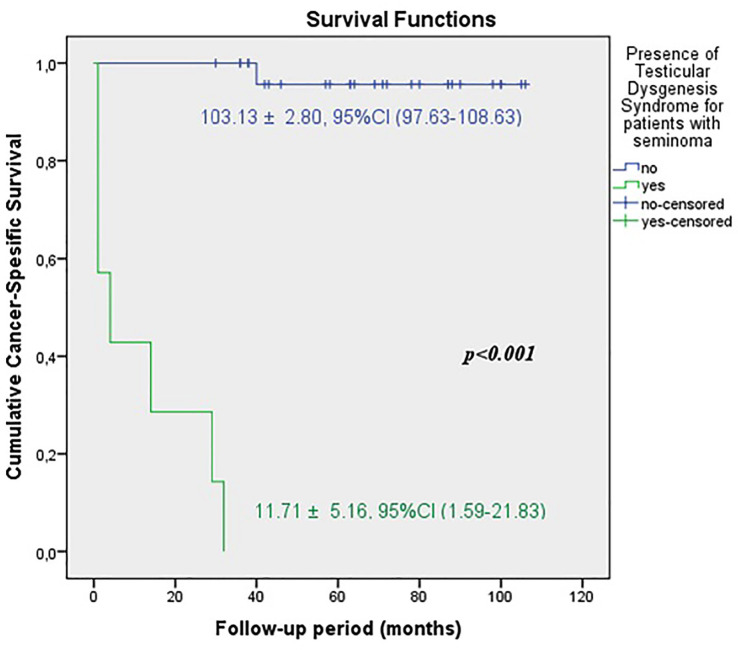
Kaplan-Meier plots of cancer-specific survival according to presence of Testicular Dysgenesis Syndrome for patients with seminoma.

**Figure 4A:**
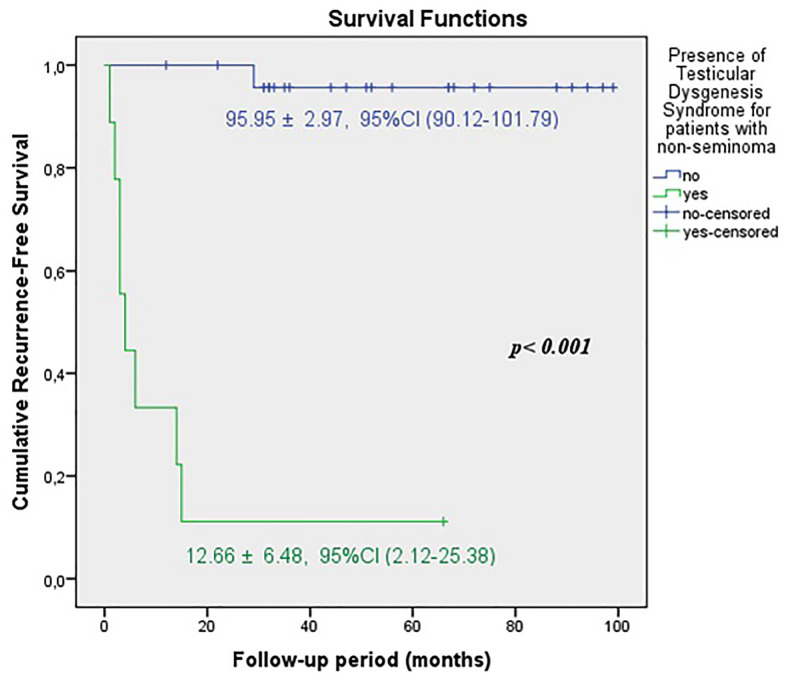
Kaplan-Meier plots of recurrence-free survival according to presence of Testicular Dysgenesis Syndrome for patients with non-seminoma.

**Figure 4B:**
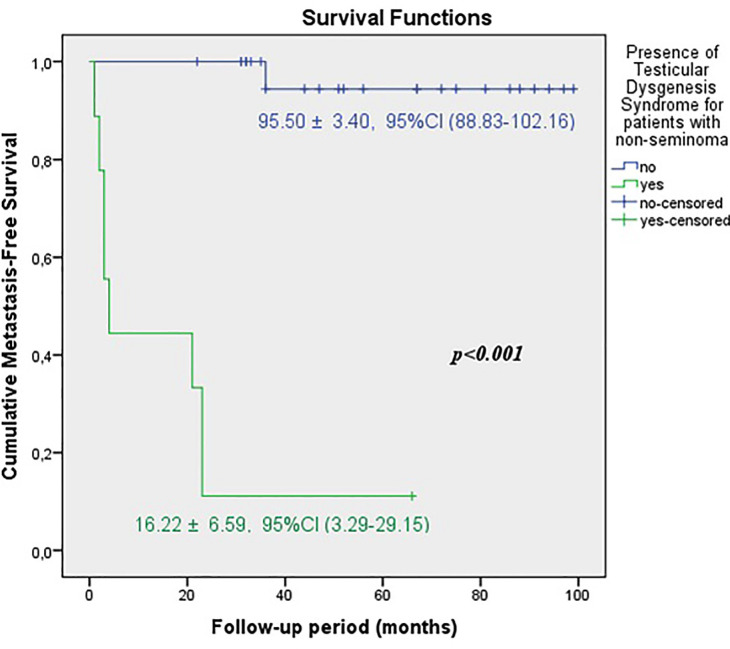
Kaplan-Meier plots of metastasis-free survival according to presence of Testicular Dysgenesis Syndrome for patients with non-seminoma.

**Figure 4C:**
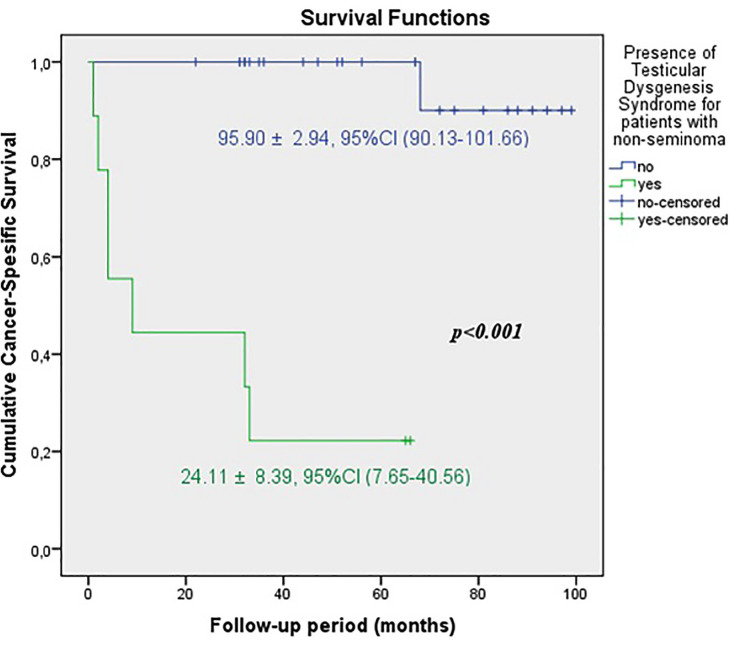
Kaplan-Meier plots of cancer-specific survival according to presence of Testicular Dysgenesis Syndrome for patients with non-seminoma.

When we evaluated the patients in early and advanced tumor stages, we found that there were no significant differences between the rates of undescended testis, hypospadias and disorders of semen parameters. However, the rate of TDS was found significantly higher in advanced stage (Table-2).

In univariate analysis, clinical stage, β-hCG, LDH, the presences of undescended testis, disorders of semen parameters, hypospadias, atrophic testis and TDS were found as independent predictive factors to estimate local recurrence, distant metastasis and cancer-specific survival (CSS). In multivariate analysis, the most important independent predictive factor was TDS to determine local recurrence, distant metastasis and cancer-specific survival RFS, MFS and CSS in both seminomas and nonseminomas as well as all patients with TGCT. In addition, clinical stage was found as a predictive factor for development of distant metastasis in all patients with TGCT (Table-4).

**Table 4:** Predictive factors for local recurrence, distant metastases and cancer-specific survival.

All patients with testicular germ cell tumor	Univariate Model				Multivariate Model			
Development of local recurrence	HR	%95 Confidence Interval		p	HR	%95 Confidence Interval		p
		Lower	Upper			Lower	Upper	
Clinical stage	12,471	2,320	34,624	**0.005**				
β-hCG	1,001	1,000	1,011	**0.003**				
LDH	1,009	1,000	1,019	**<0.001**				
Undescended testis	20,238	9,128	106,902	**<0.001**				
Disorders of semen parameters	7,250	2,359	22,281	**0.001**				
Hypospadiass	16,182	4,286	61,100	**<0.001**				
Atrophic testis	11,186	3,641	34,373	**<0.001**				
Testicular Dysgenesis Syndrome	31,911	12,414	289,130	**<0.001**	31,911	12,414	289,130	**<0.001**


All patients with testicular germ cell tumor	Univariate Model				Multivariate Model			
Development of distant metastasis	HR	%95 Confidence Interval		p	HR	%95 Confidence Interval		p
		Lower	Upper			Lower	Upper	
Clinical stage	14,988	5,575	36,668	**0.001**	12,827	4,186	36,738	**0.019**
β-hCG	1,003	1,000	1,008	**0.007**				
LDH	1,004	1,000	1,017	**0.001**				
Undescended testis	11,966	5,069	44,184	**<0.001**				
Disorders of semen parameters	12,928	5,316	41,917	**<0.001**				
Hypospadiass	11,342	3,082	41,741	**<0.001**				
Atrophic testis	11,626	4,009	33,711	**<0.001**				
Testicular Dysgenesis Syndrome	35,120	15,785	357,499	**<0.001**	35,120	15,785	357,499	**<0.001**


All patients with testicular germ cell tumor	Univariate Model				Multivariate Model			
Cancer spesific survival	HR	%95 Confidence Interval		p	HR	%95 Confidence Interval		p
		Lower	Upper			Lower	Upper	
Clinical stage	12,404	2,339	33,316	**0.003**				
β-hCG	1,002	1,000	1,016	**0.006**				
LDH	1,006	1,000	1,014	**0.001**				
Undescended testis	19,559	6,302	60,709	**<0.001**				
Disorders of semen parameters	10,602	3,729	30,143	**<0.001**				
Hypospadiass	12,398	3,317	46,342	**<0.001**				
Atrophic testis	11,661	4,000	33,994	**<0.001**				
Testicular Dysgenesis Syndrome	37,148	12,844	780,852	**<0.001**	37,148	12,844	780,852	**<0.001**


Patients with seminoma	Univariate Model				Multivariate Model			
Development of local recurrence	HR	%95 Confidence Interval		p	HR	%95 Confidence Interval		p
		Lower	Upper			Lower	Upper	
Clinical stage	12,564	0,926	7,101	**0.047**				
LDH	1,001	1,000	1,002	**0.038**				
Undescended testis	13,159	3,790	141,529	**0.001**				
Disorders of semen parameters	9,347	1,449	60,312	**0.019**				
Hypospadiass	15,641	3,555	184,944	**0.001**				
Atrophic testis	18,323	2,931	114,540	**0.002**				
Testicular Dysgenesis Syndrome	30,628	4,635	129,742	**0.001**	30,628	4,635	129,742	**0.001**


Patients with seminoma	Univariate Model				Multivariate Model			
Development of distant metastasis	HR	%95 Confidence Interval		p	HR	%95 Confidence Interval		p
		Lower	Upper			Lower	Upper	
Clinical stage	12,766	1,179	6,493	**0.019**				
β-hCG	1,005	1,001	1,010	**0.020**				
LDH	1,002	1,000	1,009	**0.016**				
Undescended testis	9,470	2,111	42,487	**0.003**				
Disorders of semen parameters	11,228	5,605	43,994	**<0.001**				
Hypospadiass	9,076	1,735	47,469	**0.009**				
Atrophic testis	13,015	2,880	58,805	**0.001**				
Testicular Dysgenesis Syndrome	44,261	5,898	411,469	**<0.001**	44,261	5,898	411,469	**<0.001**


Patients with seminoma	Univariate Model				Multivariate Model			
Cancer specific survival	HR	%95 Confidence Interval		p	HR	%95 Confidence Interval		p
		Lower	Upper			Lower	Upper	
Clinical stage	12,766	1,177	6,502	**0.020**				
β-hCG	1,000	0,997	1,007	**0.020**				
LDH	1,001	0,987	1,024	**0.018**				
Undescended testis	10,323	2,274	46,861	**0.002**				
Disorders of semen parameters	9,891	6,011	21,240	**<0.001**				
Hypospadiass	8,991	1,720	46,989	**0.009**				
Atrophic testis	14,310	3,097	66,129	**0.001**				
Testicular Dysgenesis Syndrome	49,691	2,004	338,743	**0.026**	49,691	2,004	338,743	**0.026**


Patients with non-seminoma	Univariate Model				Multivariate Model			
Development of local recurrence	HR	%95 Confidence Interval		p	HR	%95 Confidence Interval		p
		Lower	Upper			Lower	Upper	
Clinical stage	12,317	1,041	5,157	**0.039**				
LDH	1,000	0,879	1,011	**0.005**				
Undescended testis	18,411	6,143	81,928	**<0.001**				
Disorders of semen parameters	5,827	1,439	23,594	**0.014**				
Hypospadiass	2,457	1,203	4,916	**0.045**				
Atrophic testis	8,680	1,906	39,534	**0.005**				
Testicular Dysgenesis Syndrome	42,666	6,542	173,660	**<0.001**	42,666	6,542	173,660	**<0.001**


Patients with non-seminoma	Univariate Model				Multivariate Model			
Development of distant metastasis	HR	%95 Confidence Interval		p	HR	%95 Confidence Interval		p
		Lower	Upper			Lower	Upper	
Clinical stage	12,342	1,057	5,190	**0.036**				
β-hCG	1,005	1,001	1,010	**0.020**				
LDH	1,000	0,984	1,003	**0.005**				
Undescended testis	14,095	4,720	122,992	**<0.001**				
Disorders of semen parameters	6,603	1,610	27,083	**0.009**				
Hypospadiass	2,123	1,015	3,356	**0.042**				
Atrophic testis	6,456	1,538	27,102	**0.011**				
Testicular Dysgenesis Syndrome	28,536	9,458	39,177	**0.046**	28,536	9,458	39,177	**0.046**


Patients with non-seminoma	Univariate Model				Multivariate Model			
Cancer specific survival	HR	%95 Confidence Interval		p	HR	%95 Confidence Interval		p
		Lower	Upper			Lower	Upper	
Clinical stage	12,238	0,969	5,168	**0.039**				
β-hCG	1,000	0,997	1,007	**0.020**				
LDH	1,002	0,802	1,011	**0.005**				
Undescended testis	15,891	5,827	44,495	**<0.001**				
Disorders of semen parameters	4,095	0,790	21,219	**0.033**				
Hypospadiass	1,402	1,017	3,916	**0.041**				
Atrophic testis	8,372	1,849	37,908	**0.006**				
Testicular Dysgenesis Syndrome	25,634	2,479	39,951	**0.034**	25,634	2,479	39,951	**0.034**

p <0.05 Bold values indicates statistical significance.

**β-hCG** = beta human chorionic gonadotropin; **HR** = hazard ratio; **LDH** = lactate dehydrogenase Cox Regression Analysis

## DISCUSSION

Recent studies in the United States have remarked that TGCT is the most common cancer among men between the ages of 15-44 years and constitutes 98% of all testis malignancies (10). Undescended testis and hypospadias, which are the other components of TDS, affect 2-9% and 0.2-1% of male newborns, respectively (11). Approximately 10-15% of married couples have infertility and the male factor is responsible for about half of the cases (12). Although most of these disorders are assumed to be associated with TDS, further studies are needed to make the definition of TDS widely acceptable (13).

It is thought that embryonic hormonal disturbances related to androgens play a role on abnormal differentiation of primordial germ cells (14). These are usually manifested by antenatal origin. Undescended testis and hypospadias give symptoms at neonatal period whereas poor quality of semen and development of TGCT manifest after puberty (9). Animal models and epidemiological researches have revealed that deficiencies in the production of androgens, disorders of androgen receptor expression, disturbance in androgen levels, exposure to anti-androgenic or estrogenic disruptors were attributed to the pathogenesis of TDS (6, 15). These factors are blamed for causing dysfunctions and dysregulation of Leydig and Sertoli cells. As a result, disruption of testicular differentiation and development give rise to impairment of normal gonadal maturation. Consequently, irreversible testicular dysgenesis is unavoidable and it results in genital malformation (such as hypospadias and undescended testis), impaired spermatogenesis and TGCT (7). TDS is predominantly triggered by environmental exposure, genetic and lifestyle factors as well as embryonic hormonal disturbances. All of these predisposing factors similarly affect the pathophysiology of TDS.

Skakkebaek et al. (16) re-analysed 20 testicular biopsies which were derived from patients with infertility, undescended testis and hypospadias. TGCT was detected in 45% of patients. But they did not evaluate the relation between presence of TDS and TGCT prognosis. Guminska et al. (17) detected that testes with disturbed spermatogenesis were more prone to development of TGCT. They investigated morphometric analysis of seminiferous epithelium, qualitative and quantitative features of Leydig cells, seminiferous tubules diameter and thickness of tubular wall. It was shown that poor testicular histomorphological features related to testicular dysgenesis increased the incidence of TGCT but they did not worsen the tumor prognosis.

Another source that supports the biological mechanism of TDS, can be attributed to our knowledge about testicular microlithiasis (TM). TM which is detected incidentally during the scrotal ultrasound, is a rare condition. It is observed around 0.6-9.0% in symptomatic male adults and around 2.4-5.6% in asymptomatic males (17). Although the presence of TM alone is not an indication for further investigation, the presence of other risk factors carries risk for TGCT development. These risk factors include history of previous TGCT, undescended testis, orchidopexy, testicular atrophy (testicular volume <12mL) and subfertility (18). As it can be understood, the risk of TGCT increases in the presence of undescended testis and subfertility (19). From this point of view, we can think that embryological development and pathogenesis of all these disorders mentioned above is caused by a common fetal origin. This condition can be interpreted as supporting the TDS hypothesis (20).

It should be known that TDS hypothesis does not mean that all affected men develop all four components (21). A very broad variety of phenotypes can be seen in TDS. This wide spectrum ranges from genetically determined “Disorders of Sex Development” to mild forms such as slightly decreased spermatogenesis (5). One component of TDS may increase the possibility of other components’ existence. Especially, if there are more than one component, the presence of other components should be examined more carefully to detect TDS (22).

Environmental factors and genetic susceptibility are responsible for the etiology of TDS and TGCT (15). In literature, there are many animal models and epidemiological studies demonstrating this relationship (13, 15). Current animal models, involving fetal exposure to “Di-n-butyl phthalate” have been highlighting that environmental factors are most likely responsible for TDS and TGCT (4, 5). Translation of the animal model’s findings to the human biology have been linked to TDS (4). But we have found no detailed studies investigating whether TDS or its components affect the oncological outcomes of TGCT.

Cure is achievable in 95% of all patients with TGCT. At the time of diagnosis, 75-80% of seminomas are stage I. In this group, rete testis invasion and/ or tumor size larger than 4 cm are risk factors that predict relapse and occult metastasis. In the follow-up periods of seminomas after adjuvant therapy, systemic recurrence rate was 1-4%, while occult metastasis rate was 10-15%. If any adjuvant treatments are not given to the patients with risk factors, the rate of local recurrence or retroperitoneal metastasis in five years is 15-20% (23). 55% of non-seminomas are stage I at the time of diagnosis. The worst risk factor that predict relapse and occult metastasis is LVI for non-seminomas, while other important prognostic risk factors are percentage of embryonal carcinoma >50% and a proliferation rate >70%. More than 30% of them have occult metastasis at diagnosis. 70% of them can develop local recurrence if any adjuvant treatments are not performed to the patients with risk factors. In the presence of LVI, systemic relapse rate was 14-22% and occult metastasis rate was 48% (24).

The local recurrence rates were reported as 9-24% in stage IIA/B, whereas the cure rate is approximately 80% in stage IIC/III, despite the frontline and salvage chemotherapy (25). In metastatic disease, 5-year survival rates were reported by the International Germ Cell Cancer Collaboration Group to be 91% in the favorable risk group, 79% in the intermediate risk group and 48% in the poor risk group (25).

In our study, during median 57 (6-106) months follow-up in all patients, local recurrence rate was 21.7%, distant metastasis rate was 24.6%, 5-year cancer specific survival CSS rate was 76.8%. When our patients were divided into two groups as early and advanced stages, the rates of local recurrence, distant metastasis and 5-year cancer specific survival were 9.1%, 9.1%, 90.9% for early stage respectively, whereas the rates were 33.3%, 38.9% and 63.9% for advanced stage. The duration of recurrence-free survival RFS (96.57 ± 4.65 months), metastasis-free survival MFS (96.99 ± 4.43 months), cancer-specific survival CSS (96.67 ± 4.57 months) were observed significantly higher in early stage. Although our survival rates are less than the rates in the current literature (26), this may be explained by the small patients populations and short follow-up periods.

Undescended testis is known to be an important risk factor for the development of TGCT. The relative risk of TGCT was 2.23 even if patients underwent orchiopexy before 13 years old (27). Moirano et al. (28) observed that undescended testis was higher in TGCT group (11.4%) than in healthy control group (3.0%). Hanson et al. (29) detected an increased risk of testicular cancer (hazard rate of 3.3) in subfertile men when compared with fertile men (29). In addition, hypospadias was found associated with an increased relative risk for TGCT development (hazard rate of 2.13) (30).

We could not evaluate whether undescended testis, disorders of semen parameters and hypospadias were risk factors for the development of TGCT because we did not have a healthy control group. We compared these three components in terms of tumor stages. When these components were analyzed individually, we did not find significantly differences between early and advanced stage groups. But the rate of TDS was significantly higher in patients with advanced stage. This finding suggested that even if the components alone did not contain poor prognostic features for TGCT development, a significant increase was observed in tumor stages in the patients diagnosed with TDS (having more than one component).

In subgroup analysis, we divided patients into two groups according to presence of TDS. Although the small numbers of patients in the TDS group decreased the statistical power of the study, we found significantly higher rates of local recurrence (75% vs. 5.7), distant metastasis (93.6% vs 3.8%) and cancer related mortality (87.5% vs. 3.8%) in TDS group rather than those without TDS. When we evaluated two different tumor types separately, in the presence of TDS, the rate of local recurrence (88.9% vs. 57.1%) was higher in non-seminomas; whereas distant metastasis (100% vs. 88.9%) and cancer-specific mortality rates (100% vs. 77.8%) were higher in seminomas. It is obvious that these findings will be more reliable when a much larger patient population is evaluated.

To the best of our knowledge, this is the first study to evaluate the prognostic value of TDS components on TGCT prognosis and oncological outcomes. However, this study has some limitations. The main limitations of our study are retrospective, non randomized design with small patient population in a single center. Future studies that have larger numbers of patients with multicentre, prospective, randomized, controlled, long-term follow-up are needed to verify our results and explain more new details about this hypothesis, especially for the subgroup analysis with patients having TDS. We presented our findings as “Preliminary Results” because it was not easy to have comprehensive results due to small patient population and relatively short follow-up. Since this topic has not been studied before, we think that our findings as “Preliminary Results” may be a step for further studies.

## CONCLUSIONS

In conclusion, although there have been many controversial views on TDS since the last two decades, most studies have shown the relationship between the four components of TDS. We observed the fact that TDS was detected to be higher in advanced stages of TGCT. Moreover, we have seen a significant increase in the rates of local recurrence, distant metastasis and cancer specific mortality in the presence of TDS.

## Abreviaturas

AFP: Alpha-fetoprotein;
β-hCG: Beta human chorionic gonadotropin;
LDH: Lactate dehydrogenase;
LVI: lymphovascular invasion;
TDS: Testicular Dysgenesis Syndrome;
TGCT: Testicular germ cell tumor;
TM: Testicular microlithiasis.

## References

[1] 1. Skakkebaek NE, Rajpert-De Meyts E, Main KM. Testicular dysgenesis syndrome: an increasingly common developmental disorder with environmental aspects. Hum Reprod. 2001;16:972-8. 10.1093/humrep/16.5.972 11331648

[2] 2. Jørgensen N, Asklund C, Carlsen E, Skakkebaek NE. Coordinated European investigations of semen quality: results from studies of Scandinavian young men is a matter of concern. Int J Androl. 2006;29:54-61; discussion 105-8. 10.1111/j.1365-2605.2005.00635.x 16466524

[3] 3. Sharpe RM, Skakkebaek NE. Testicular dysgenesis syndrome: mechanistic insights and potential new downstream effects. Fertil Steril. 2008;89(2 Suppl):e33-8. 10.1016/j.fertnstert.2007.12.026 18308057

[4] 4. van den Driesche S, Kilcoyne KR, Wagner I, Rebourcet D, Boyle A, Mitchell R, et al. Experimentally induced testicular dysgenesis syndrome originates in the masculinization programming window. JCI Insight. 2017;2:e91204. 10.1172/jci.insight.91204 PMC5358493 28352662

[5] 5. Jørgensen N, Rajpert-De Meyts E, Main KM, Skakkebaek NE. Testicular dysgenesis syndrome comprises some but not all cases of hypospadias and impaired spermatogenesis. Int J Androl. 2010;33:298-303. 10.1111/j.1365-2605.2009.01050.x 20132348

[6] 6. van den Driesche S, Kolovos P, Platts S, Drake AJ, Sharpe RM. Inter-relationship between testicular dysgenesis and Leydig cell function in the masculinization programming window in the rat. PLoS One. 2012;7:e30111. 10.1371/journal.pone.0030111 PMC3256232 22253897

[7] 7. Söder O. Sexual dimorphism of gonadal development. Best Pract Res Clin Endocrinol Metab. 2007;21:381-91. 10.1016/j.beem.2007.05.002 17875486

[8] 8. Akre O, Richiardi L. Does a testicular dysgenesis syndrome exist? Hum Reprod. 2009;24:2053-60. 10.1093/humrep/dep174 19443456

[9] 9. Spiller CM, Bowles J. Germ cell neoplasia in situ: The precursor cell for invasive germ cell tumors of the testis. Int J Biochem Cell Biol. 2017;86:22-25. 10.1016/j.biocel.2017.03.004 28288913

[10] 10. McGlynn KA, Cook MB. Etiologic factors in testicular germcell tumors. Future Oncol. 2009;5:1389-402. 10.2217/fon.09.116 PMC3000220 19903067

[11] 11. Toppari J, Virtanen HE, Main KM, Skakkebaek NE. Cryptorchidism and hypospadias as a sign of testicular dysgenesis syndrome (TDS): environmental connection. Birth Defects Res A Clin Mol Teratol. 2010;88:910-9. 10.1002/bdra.20707 20865786

[12] 12. Erdemir F, Firat F, Gençten Y. The Evaluation and Clinical Significance of Sperm Morphology. Turk Urol Sem. 2011;2:11-7.

[13] 13. Rajpert-De Meyts E. Developmental model for the pathogenesis of testicular carcinoma in situ: genetic and environmental aspects. Hum Reprod Update. 2006;12:303-23. 10.1093/humupd/dmk006 16540528

[14] 14. Baskin LS. Hypospadias and urethral development. J Urol. 2000;163:951-6. 10688029

[15] 15. Xing JS, Bai ZM. Is testicular dysgenesis syndrome a genetic, endocrine, or environmental disease, or an unexplained reproductive disorder? Life Sci. 2018;194:120-129. 10.1016/j.lfs.2017.11.039 29183799

[16] 16. Skakkebaek NE, Holm M, Hoei-Hansen C, Jørgensen N, Rajpert-De Meyts E. Association between testicular dysgenesis syndrome (TDS) and testicular neoplasia: evidence from 20 adult patients with signs of maldevelopment of the testis. APMIS. 2003;111:1-9; discussion 9-11. 10.1034/j.1600-0463.2003.11101031.x 12752226

[17] 17. Guminska A, Oszukowska E, Kuzanski W, Sosnowski M, Wolski JK, Walczak-Jedrzejowska R, et al. Less advanced testicular dysgenesis is associated by a higher prevalence of germ cell neoplasia. Int J Androl. 2010;33:e153-62. 10.1111/j.1365-2605.2009.00981.x 19719533

[18] 18. Balawender K, Orkisz S, Wisz P. Testicular microlithiasis: what urologists should know. A review of the current literature. Cent European J Urol. 2018;71:310-314. 10.5173/ceju.2018.1728 PMC6202617 30386652

[19] 19. Pedersen MR, Rafaelsen SR, Moller H, Vedsted P, Osther PJ. Testicular microlithiasis and testicular cancer: review of the literature. Int Urol Nephrol. 2016;48:1079-86. 10.1007/s11255-016-1267-2 27007613

[20] 20. Dantsev IS, Ivkin EV, Tryakin AA, Godlevski DN, Latyshev OY, Rudenko VV, et5 al. Genes associated with testicular germ cell tumors and testicular dysgenesis in patients with testicular microlithiasis. Asian J Androl. 2018;20:593-599. 10.4103/aja.aja_54_18 PMC6219295 30027931

[21] 21. Mahood IK, Hallmark N, McKinnell C, Walker M, Fisher JS, Sharpe RM. Abnormal Leydig Cell aggregation in the fetal testis of rats exposed to di (n-butyl) phthalate and its possible role in testicular dysgenesis. Endocrinology. 2005;146:613-23. 10.1210/en.2004-0671 15539561

[22] 22. Hoei-Hansen CE, Sommer P, Rajpert-De Meyts E, Skakkebaek NE. A rare diagnosis: testicular dysgenesis with carcinoma in situ detected in a patient with ultrasonic microlithiasis. Asian J Androl. 2005;7:445-7. 10.1111/j.1745-7262.2005.00020.x 16281095

[23] 23. Shelley MD, Burgon K, Mason MD. Treatment of testicular germ-cell cancer: a cochrane evidence-based systematic review. Cancer Treat Rev. 2002;28:237-53. 10.1016/s0305-7372(02)00059-2 12435371

[24] 24. Ondrusova M, Suchansky M, Psota M, Zeleny T, Ondrus D. Late relapse in stage I of nonseminomatous germ cell testicular cancer on surveillance. Bratisl Lek Listy. 2018;119:3-5. 10.4149/BLL_2018_001 29405722

[25] 25. Adra N, Einhorn LH. Testicular cancer update. Clin Adv Hematol Oncol. 2017;15:386-396. 28591093

[26] 26. Withington J, Cole AP, Meyer CP, Seisen T, Schmid M, Lipsitz SR, et al. Comparison of testis cancer-specific survival: an analysis of national cancer registry data from the USA, UK and Germany. BJU Int. 2019;123:385-387. 10.1111/bju.14616 30536825

[27] 27. Pettersson A, Richiardi L, Nordenskjold A, Kaijser M, Akre O. Age at surgery for undescended testis and risk of testicular cancer. N Engl J Med. 2007;356:1835-41. 10.1056/NEJMoa067588 17476009

[28] 28. Moirano G, Zugna D, Grasso C, Mirabelli D, Lista P, Ciuffreda L, et al. Postnatal risk factors for testicular cancer: The EPSAM case-control study. Int J Cancer. 2017;141:1803-1810. 10.1002/ijc.30884 28699204

[29] 29. Hanson HA, Anderson RE, Aston KI, Carrell DT, Smith KR, Hotaling JM. Subfertility increases risk of testicular cancer: evidence from population-based semen samples. Fertil Steril. 2016;105:322-8.e1. 10.1016/j.fertnstert.2015.10.027 PMC4744156 26604070

[30] 30. Schnack TH, Poulsen G, Myrup C, Wohlfahrt J, Melbye M. Familial coaggregation of cryptorchidism, hypospadias, and testicular germ cell cancer: a nationwide cohort study. J Natl Cancer Inst. 2010;102:187-92. 10.1093/jnci/djp457 20026812

